# A Cellulose Ionogel with Rubber-Like Stretchability for Low-Grade Heat Harvesting

**DOI:** 10.34133/research.0533

**Published:** 2024-11-18

**Authors:** Qian Long, Geyuan Jiang, Jianfei Zhou, Dawei Zhao, Haipeng Yu

**Affiliations:** ^1^ Key Laboratory on Resources Chemicals and Materials of Ministry of Education, Shenyang University of Chemical Technology, Shenyang, China.; ^2^ College of Biomass Science and Engineering, Sichuan University, Chengdu, China.; ^3^ Key Laboratory of Bio-based Material Science and Technology of Ministry of Education, Northeast Forestry University, Harbin, China.

## Abstract

Achieving rubber-like stretchability in cellulose ionogels presents a substantial challenge due to the intrinsically extended chain configuration of cellulose. Inspired by the molecular configuration of natural rubber, we address this challenge by using cyanoethyl as a substitute for 1.5 hydroxyl on the D-glucose unit of cellulose. This strategy innovatively triggers the transformation of cellulose molecules into a coiled chain configuration, facilitating the creation of an ultra-stretchable ionogel free from any petrochemical polymers. The resultant ionogel demonstrates mechanical ductility comparable to that of a rubber band, achieving an elongation strain of nearly 1,000% while maintaining a tensile strength of up to 1.8 MPa and exhibiting a biomodulus akin to that of human skin, recorded at 63 kPa. Additionally, this stretchable ionogel presents skin-like self-healing behavior, favorable biocompatibility, and noteworthy thermoelectric properties, highlighted by a Seebeck coefficient of approximately 68 mV K^−1^. This study delineates a feasible molecular approach for developing stretchable ionogels from biomass resources, potentially revolutionizing self-powered stretchable electronics for integration with human tissues and skin.

## Introduction

Ionogels, composed of cross-linked polymer networks and ionic pairs, are flexible with ionic conductivity [1,2], rendering them increasingly relevant in applications such as electronic skins (e-skins) [3], flexible electronics [4], and human–machine interactions [5]. Recently, the stretchability of ionogels has attracted considerable attention [6–8]. This is attributed to its essential role in providing a seamless interface with biological tissues and enabling the detection of high-fidelity signals [9–11]. However, most network molecules in stretched ionogels are sourced from petrochemical polymers, such as polyacrylamide [6], polydimethylsiloxane [12], and polyurethane [5]. Environmental and sustainability issues related to non-biodegradable petrochemical polymers have underscored the urgent need to develop sustainable, stretchable ionogels using biomass macromolecules as the structural framework [13,14].

Cellulose, a natural polysaccharide polymer widely found in plants [15–18], presents a viable alternative [19–21]. Each glucose unit in a cellulose macromolecule contains 3 hydroxyl (-OH) groups, which are conducive to forming a hydrogen bond (H-bond) network and facilitating structural substitution modifications for molecular configuration design. Innovative cellulosic ionogels, such as those with dynamic and self-regulating properties, have been developed through the molecular-scale modulation of the H-bond network, demonstrating excellent mechanical strength and switchable performance [3,22,23]. Nevertheless, achieving cellulose ionogels that present stretchability without relying on petrochemical polymers remains challenging due to the extended chain configuration of cellulose molecules [24].

**Fig. 1.**
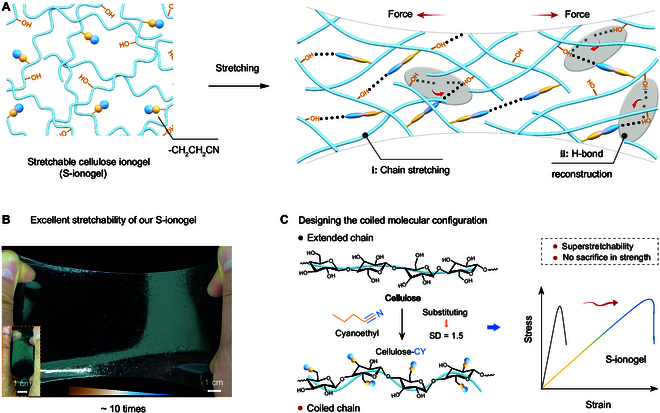
Super-stretchable cellulose ionogel (S-ionogel) derived from a molecular configuration design. (A) Schematic diagram of stretchable molecular networks of S-ionogel. (B) Optical images of the S-ionogel showing the hyper-stretchable behavior. (C) Development of the coiled molecular configuration of S-ionogel. The right panel shows the mechanical properties of S-ionogels with an SD of 1.5.

In this context, the molecular structure of natural rubber merits attention. The random coil conformation of the rubber molecule imparts its inherent stretchability. The vulcanization modification—strengthening intermolecular cross-linking—further augments the mechanical strength of rubber materials [25]. Drawing inspiration from the natural rubber, we develop a kind of super-stretchable cellulose ionogel (S-ionogel, Fig. 1A). This is accomplished through a molecular curling configuration design that partially replaces the -OH groups in cellulose with cyanoethyl groups (-CH_2_CH_2_CN). The resultant S-ionogel displays promising stretchability with a tensile strain of more than 990%, nearly 10-fold its original length (Fig. 1B and Movie S1); additionally, it also presents a high ionic conductivity of 21.35 mS cm^−1^ and a thermal gradient ratio of around 68 mV K^−1^, marking the highest levels of both extensibility and thermoelectric properties recorded in cellulose ionogels.

Moreover, when the substitution degree (SD) reaches 1.5—achieving an equal ratio of -OH groups to -CH_2_CH_2_CN groups within the cellulose chain (forming the cellulose-cyanoethyl molecule with optimal curled state, noted as cellulose-CY)—the S-ionogel retains not only exceptional stretchability but also robust mechanical strength (Fig. 1C). The dual capability, maintaining mechanical strength while enhancing extensibility, stands out in comparison to other stretchable gels [7,26,27], which typically sacrifices mechanical strength for increased extensibility. This mechanical performance of our S-ionogel stems from its coiled molecular configuration and the reconstruction of H-bonds between -OH groups and -CH_2_CH_2_CN groups as the cellulose chains are stretched (right panel of Fig. 1A).

## Results

### Mechanical properties of S-ionogel

Cellulose ionogels, noted for their flexibility and ionic conductivity, are increasingly acknowledged as prospective soft materials for applications in e-skins and flexible electronics [28–30]. However, due to the structural vulnerabilities of cellulose ionogels under external forces (Fig. S1), they struggle to exhibit stretchability comparable to natural rubber and petrochemical polymer hydrogels. We are pleased to report the development of a stretchable cellulose ionic gel, termed S-ionogel. This ionic gel modifies the molecular configuration by substituting the -OH groups in cellulose with -CH_2_CH_2_CN groups (see Fig. S2). The resulting coiled molecular chain not only enhances the stretchability of S-ionogel but also improves its interface stability with biological tissues. Therefore, the S-ionogel represents a promising alternative to petrochemical polymer hydrogels or ionic gels for developing stretchable electronics.

**Fig. 2.**
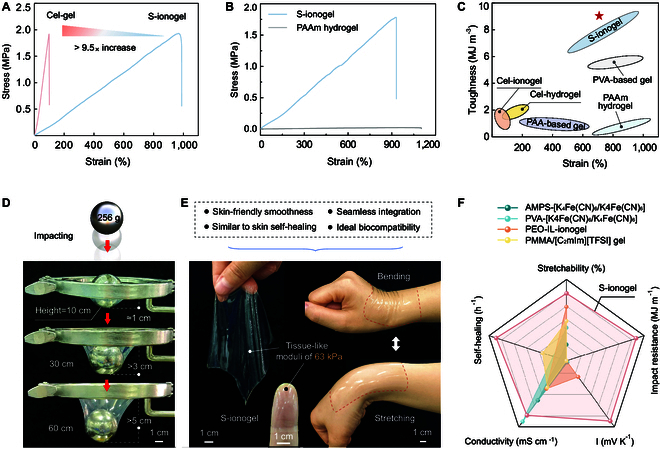
Investigating the mechanical properties of S-ionogel. (A) Tensile stress–strain curves of our S-ionogel and traditional Cel-gel. (B) Comparing the stretching behavior of S-ionogel and petrochemical PAAm hydrogel. (C) The Ashby diagram of toughness and elongation of S-ionogel and familiar polymeric complexes. (D) Free-fall impact resistance of the S-ionogel. The weight of the iron ball is 256 g. (E) Optical images of the S-ionogel exhibiting flexibility, tissue resilience, and seamless integration with the human wrist and finger. (F) The radar plot showing the key properties of S-ionogel compared with other reported gel materials.

When the SD reaches 1.5, i.e., this represents a critical point where the proportion of -CH_2_CH_2_CN and -OH groups in the cellulose chain is approximately equal (Fig. S3). At this juncture, the S-ionogel attains optimal extensibility. Specifically, it achieves a tensile strain of 998.23% (Fig. 2A), surpassing the traditional cellulose ionogel (Cel-gel) by more than 9.5 times. Notably, this remarkable increase in stretchability did not compromise the mechanical strength of the S-ionogel, which remained robust under extensive deformation. This breakthrough has the potential to revolutionize the application of cellulose ionogels in demanding bioelectronics fields, where both high elasticity and material strength are crucial.

Like natural rubber, our ionogels demonstrate the elastic deformation within a 0% to 100% strain range—stretching and lengthening followed by a recovery to their original shape upon the cessation of force (Fig. S4). Remarkably, the mechanical properties of S-ionogel are considerably enhanced after undergoing a pre-stretching treatment to about 800% strain, resulting in a tensile strength exceeding 6 MPa (Fig. S5). This phenomenon indicates that the S-ionogel initially exhibited rubber-like elastomeric deformation; as the stretching force increased continuously, its mechanical strengthening and plastic deformation properties are activated. This characteristic of S-ionogel closely mimics human skin, allowing it to absorb and withstand external forces without sustaining damage.

We then compared S-ionogel with the widely recognized stretchable polyacrylamide (PAAm) hydrogel to better highlight its mechanical stretchability. As demonstrated in Fig. 2B, the extensibility of S-ionogel is comparable to the PAAm hydrogel, and it also demonstrates a considerably higher tensile strength of 1.78 MPa, which is over 77 times that of the PAAm hydrogel (0.023 MPa). Additionally, the S-ionogel also displays exceptional toughness, achieving an impressive rate of 9.015 MJ m^−3^. It outstrips numerous other stretchable petrochemical gels [17,31–33], including polyvinyl alcohol-based gel, polyacrylic acid-based gel, and PAAm hydrogel (Fig. 2C).

Strikingly, the S-ionogel also demonstrates a promising energy-absorbing behavior by resolving strain when subjected to external shocks, making its entire structure tougher and less prone to cracking. When a 256-g iron ball is dropped from heights of 10, 30, and 60 cm onto the S-ionogel, it securely captures the ball without any rebound, effectively dissipating the impact force through increased strain (Fig. 2D and Movie S2). This is in stark contrast to the marked damage observed in the traditional Cel-gel when subjected to the impact of the same iron ball dropped from a height of 25 cm (Fig. S6). The impact strength of our ionogel, quantified at up to 4.493 MJ m^−1^ (Fig. S7), surpasses that of other reported gel materials [17,34], highlighting its advanced properties in mechanical shock absorption.

In addition to exceptional mechanical stretchability and robustness, our S-ionogel has notable transparency, maintaining a stable transmittance exceeding 92% (Fig. S8). Moreover, it possesses self-healing capabilities, regaining structural integrity at 85 °C within 20 min (Fig. S9). The S-ionogel mimics the smoothness and resilience of human skin, evidenced by a storage modulus (*G*′) greater than the damage modulus (*G*″) (Fig. S10), and an elastic modulus measured at 63 kPa, reflecting a texture similar to that of human skin (Fig. 2E). Even under substantial bending and stretching, the S-ionogel still seamlessly integrates with human wrist and finger (Movie S3), ensuring accurate and reliable signal transmission.

The biocompatibility and non-toxicity of our S-ionogel are crucial for its integration with biological tissues. We confirmed its good biocompatibility with human skin tissues; no itching or inflammatory allergic reactions are observed after 6 h of direct contact (Fig. S11). Additionally, the human primary fibroblast (HSF cells) exposed to a high-concentration S-ionogel environment (100 mg ml^−1^) for 24 h still retains considerable cellular activity (Fig. S12). Compared to other reported stretchable gels [19,35–37], our S-ionogel presents its advantages in mechanical stretchability, toughness, self-healing, ionic conductivity, and thermoelectric properties (Fig. 2F).

### Molecular curling and mechanism of super-stretchability

**Fig. 3.**
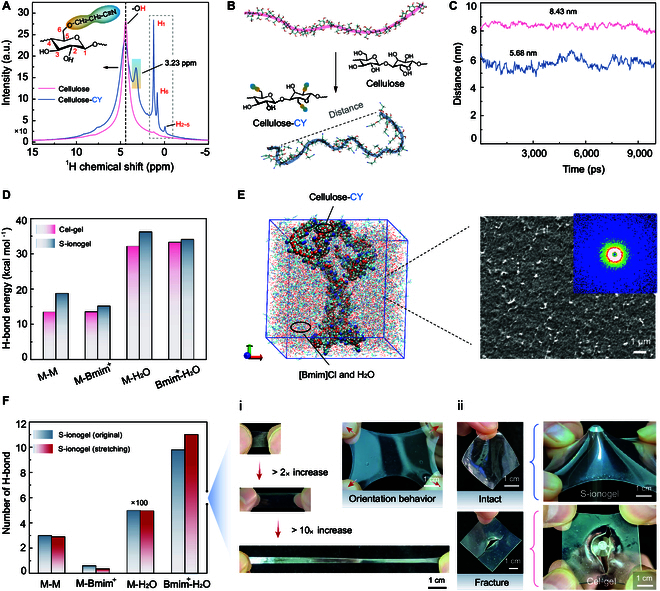
Molecular mechanism and microstructure characterization of S-ionogel. (A) Solid ^1^H-NMR spectra highlighting the molecular structure of cellulose-CY. (B) MD simulations of the overlapping chain structure of cellulose and cellulose-CY. (C) The investigation of the end-to-end distance of cellulose and cellulose-CY molecules. Pink represents the cellulose chain, and blue represents the cellulose-CY chain. (D) Comparison of the role and energy of H-bonds in Cel-gel and our S-ionogel. (E) MD snapshots, the SEM image, and the 2D scattering SAXS pattern of S-ionogel. (F) Comparing the number of H-bonds in S-ionogel before and after stretching. (i) displays the optical images of S-ionogel during the stretching process, illustrating various stretchability modes. Optical images (ii) show the puncture resistance behavior.

The molecular structure of cellulose-CY within S-ionogel is characterized using solid-state ^1^H nuclear magnetic resonance (^1^H-NMR), as shown in Fig. 3A. Both cellulose and cellulose-CY show the absorption peaks at chemical shifts of 4.41 and 4.50 parts per million (ppm), 1.22 and 1.241 ppm, 0.847 and 0.82 ppm, –0.057 and –0.021 ppm. These peaks are indicative of the hydroxyl hydrogen (-OH), C_1_ hydrogen (H_1_), C_6_ hydrogen (H_6_), and C_2_-C_5_ hydrogen (H_2_-H_5_) within the structural units of cellulose. Notably, the cellulose-CY exhibits sharper and more intense peaks than those of unmodified cellulose (Fig. 3A). This enhancement in the peak characteristics is attributed to the increased flexibility and deformation of the cellulose-CY molecular chain, facilitated by the incorporation of the -CH_2_CH_2_CN group.

Moreover, in comparison to the ^1^H-NMR spectrum of plain cellulose, the cellulose-CY displays a distinct downfield shift for the peaks corresponding to -OH and H_1_, along with an upfield shift for the absorption peaks of H_6_ and H_2_-H_5_ (Fig. 3A). These shifts result from the introduction of the -CH_2_CH_2_CN group, which increase the electron cloud density around the respective carbon atoms in the cellulose-CY. This increase reduces the cellulose-CY’s polarity and enhances steric hindrance, leading to a subsequent change in the spatial configuration of the molecular chains. These spectral changes suggest that the cyanation process primarily affected the -OH groups in cellulose, with the -OH at the C_6_ position being particularly prone to such cyanoethylation reactions.

Molecular dynamics (MD) simulations are employed to further investigate the molecular configuration behavior of cellulose before and after cyanidation (Fig. S13). Figure 3B illustrates that in its natural state, the cellulose molecule adopts a characteristic extended-chain configuration. This is quantitatively supported by measurements indicating an end-to-end distance of 8.43 nm (Fig. 3C) and a radius of gyration of 2.6 nm (Fig. S13a). These structural features explain the lack of extensibility observed in Cel-gels, which is typically found in rubber and other petrochemical-based polymer gels.

There is a notable transformation in the molecular configuration of cellulose-CY. As illustrated in Fig. 3B and S13B, the cellulose-CY molecule exhibits a wavy and curled chain configuration. The end-to-end distance shortens to 5.68 nm (Fig. 3C) while the radius of gyration decreases to 2.2 nm (Fig. S13A). From the x-ray photoelectron spectroscopy, the C-O-C binding energy in the cellulose-CY molecular backbone is 286.03 eV, higher than the 285.59 eV found in cellulose (Fig. S14A and B). This indicates a decrease in the electron cloud density of β-1,4-glycosidic bonds of cellulose-CY, making it more prone to adopting a curled configuration. This specific curled chain configuration endows the S-ionogel with excellent stretchability, rivaling that of petrochemical polymers.

Further MD analysis reveals that the curled configuration of cellulose-CY molecule increases the intermolecular distance. This change leads to a reduction in the number of H-bonds among cellulose-CY molecules compared to unmodified cellulose molecules. However, this configuration facilitates the formation of the H-bonding network between cellulose-CY and H_2_O, as evidenced by an increased number of H-bonds (Fig. S13C to E). This dual effect—the reduction in H-bonds among cellulose-CY molecules paired with the increased H-bonding with water—endows the S-ionogel with elastic properties akin to human skin. Additionally, in the S-ionogel, the H-bond energy between cellulose molecules (M), between M and [Bmim]^+^, and between M and H_2_O, as well as between [Bmim]^+^ and H_2_O, is higher than that found in traditional Cel-gels (Fig. 3D). This enhanced H-bond contributes to the formidable mechanical strength and superior structural integrity of S-ionogel during stretching.

Compared to the snapshots from MD simulations of Cel-gel (Fig. S15), the S-ionogel exhibits more curled cellulose-CY molecular aggregates (Fig. 3E). This structural characteristic endows the S-ionogel with a coherent micromorphology, characterized by a distinctive pore structure observed in the scanning electron microscopy (SEM) analysis (Fig. 3E). Additionally, the small-angle x-ray scattering (SAXS) analysis (Fig. S15) and the 2-dimensional (2D) scattering SAXS patterns (Fig. 3E) confirm that the S-ionogel, like the Cel-gel, exhibits a homogeneous molecular assembly structure. This indicates that the stretchability of S-ionogel was not limited by force orientation.

Additionally, we discover that the S-ionogel retains the stable H-bonding types, numbers, and energy (with slight variations, Fig. S17) during the stretching state (Fig. 3F). This finding indicates a dynamic equilibrium where H-bonds continuously broke and reformed within the S-ionogel during stretching. This dynamic equilibrium of H-bond allows the S-ionogel to elongate up to 10 times its initial length. This demonstrates the superior orientation behavior during elongation (Fig. 3F, i), providing significant resistance to mechanical damage and preserving its structural integrity even when subjected to stimulation from metal tips (Fig. 3F, ii). In contrast, the Cel-gel experienced severe structural damage under similar conditions (Fig. 3F, ii).

### Self-powered and stretchable e-skin from S-ionogel

**Fig. 4.**
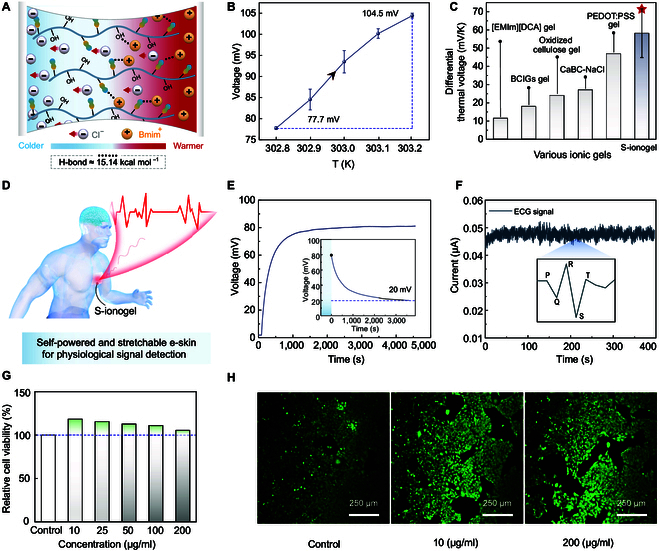
Self-powered and stretchable e-skin based on the S-ionogel. (A) The schematic diagram of the thermoelectric effect mechanism of the S-ionogel. (B) The function diagram of voltage and temperature of S-ionogel. (C) Comparison of ionic differential thermal voltage of S-ionogels with reported ionic gels. (D) The diagram of self-powered e-skin for signal detection. (E) The charging and discharging behavior of e-skin at Δ*T* of 16 ± 1 °C. (F) The monitoring of the human electrocardiograph (ECG) signals using S-ionogel-based e-skin. The inset shows an enlarged curve of one cardiac cycle. (G) The investigation of the relative cell (HaCat cells) viability of S-ionogel by the extracting method. (H) Live staining confocal images of skin tissue cells (HaCat cells) co-cultured with different ambient concentrations of S-ionogel for 24 h.

Hereafter,having identified the intrinsic mechanisms of stretchability, we return to the S-ionogel to further explore its superior properties. Comprising more than 30 wt% H_2_O and over 40 wt% ions (Fig. S18), the S-ionogel exhibits a high ionic conductivity, surpassing 10 mS cm^−1^. Within S-ionogel, the cellulose-CY molecule engages in different H-bond interactions with [Bmim]^+^ and Cl^−^. Specifically, the H-bond energy between cellulose-CY and [Bmim]^+^ is quantified at 15.14 kcal mol^−1^, whereas it forms weaker interactions with Cl^−^. This disparity in interaction strength establishes a gradient distribution of positive and negative charges within the S-ionogel when subjected to thermal stimulation, thereby enhancing the conversion of low-grade thermal energy into electrical energy (Fig. 4A). As shown in Fig. 4B, when exposed to a temperature range from 302.8 to 303.2 K, the S-ionogel shows an increase in open-circuit voltage from 77.7 to 104.5 mV (Fig. 4B). Moreover, under a modest thermal gradient (Δ*T*) of 0.55 K, the S-ionogel generates a voltage of 26.8 mV (Fig. S19A). Compared to other reported ionic gels [38–42], the S-ionogel presents an excellent differential thermal voltage of 58.19 ± 9.45 mV K^−1^ (Fig. 4C), highlighting its potential for energy harvesting.

The S-ionogel with outstanding stretchability and thermoelectric properties showcases the promising application in self-powered, stretchable e-skin for signal detection (Fig. 4D). These qualities are not present in other cellulose gel materials [43–45]. The charging and discharging behaviors of the S-ionogel when applied to human skin are depicted in Fig. 4E. The S-ionogel could charge up to approximately 80 mV, maintaining a stable output for over 3,500 s. The self-discharge curve for S-ionogel also reveals a sustainable operating voltage of 20 mV after a self-discharge period of 4,000 s (inset in Fig. 4E). Such durability can be attributed to the selective adsorption of ions by the cyanated cellulose molecules within the S-ionogel. Remarkably, the S-ionogel-based e-skin is sensitive to various stimuli, including heart vibrations (illustrated by the electrocardiogram in Fig. 4F), touch (Fig. S19B), and temperature changes (Fig. S19C). The S-ionogel-based e-skin demonstrates outstanding biocompatibility with human skin tissue, as evidenced by the relative cell activity exceeding 100% (Fig. 4G and H). Owing to its self-powering capabilities, ability to recognize specific stimuli, and superior tissue biocompatibility, this S-ionogel-based e-skin demonstrates significant potential in medical health and stretchable bioelectronics.

## Conclusion

In summary, we report a scalable molecular strategy for creating a stretchable ionogel by partially substituting the -OH groups in cellulose with -CH_2_CH_2_CN groups. This modification of functional groups transforms the extended chain configuration of cellulose into a curled one, imbuing the resulting ionogel with rubber-like super-stretchability, capable of reaching an elongation strain of nearly 1,000%. Notably, this exceptional stretchability does not compromise the mechanical strength of the ionogel. Additionally, the ionogel features a comfortable mechanical modulus of 63 kPa, high ionic conductivity at 21.35 mS cm^−1^, excellent biocompatibility, and outstanding thermoelectric properties with a Seebeck coefficient of 67.64 mV K^−1^—surpassing those of existing ionogels. This stretchable cellulose ionogel can convert human body heat directly into electricity, demonstrating a key application and serving as a self-powered, flexible e-skin that is capable of sensing cardiac, tactile, and temperature signals. This advancement in using biomass-sourced stretchable ionogels sets a promising foundation for developing sustainable, functional materials as alternatives to petrochemical polymers, holding significant potential benefits for environmental sustainability and carbon emission reduction.

## Materials and Methods

### Chemicals

Cellulose (derives from wood powder with an average degree of polymerization of ≈1,500, a crystallinity of ≈64%, and a diameter of 50 to 200 μm) and the ionic liquid of 1-butyl-3-methylimidazolium chloride ([Bmim]Cl) were obtained by reported methods [1]. Chemicals such as tetramethylammonium chloride (AR), sodium hydroxide (NaOH, AR), acrylonitrile (AR), ethyl alcohol (AR), 1-methylimidazole (99%, AR), and 1-chlorobutane (99.8%, AR) were purchased from Aladdin Reagent Co. Ltd. based in Shanghai.

### Construction of the cyanate cellulose

Dried cellulose (an average molecular weight of ≈2.42 × 10^5^) of 6 g was activated with 4 ml of aqueous solution of tetramethylammonium chloride (10 wt%) for 5 min. Then, the saturated cellulose pulp was added to 140 ml of acrylonitrile, and 4 ml of NaOH (36 wt%) solution was slowly dripped while stirring for 1.5 to 2 h at room temperature of 25 °C. After the reaction is completed, the solution was slowly dripped into an ethanol solution to obtain the cellulose with cyanoethyl (noted as cellulose-CY). Then, the cellulose-CY was washed with ethanol followed by distilled water (alternating 5 times), and we finally prepared clean cellulose-CY by drying it in an oven at 85 °C for 24 h.

### Fabrication of the stretchable cellulose ionogel

[Bmim]Cl ionic liquid (30 g) was prepared by mechanical stirring of 1-methylimidazole and 1-chlorobutane for 8 h at temperature of 80 °C. Then, the dried cellulose-CY of 1.579 g was added into [Bmim]Cl at 85 °C for 4 h to physically dissolve cellulose-CY, resulting in a homogeneous cellulose-CY molecule/[Bmim]Cl system with a cellulose-CY content of around 5 wt%. The cellulose-CY colloid was obtained after the homogeneous system was placed on the glass sheet in a vacuum drying oven (80 °C and vacuumed at –0.1 MPa) for 24 h. Finally, we obtained the stretchable cellulose ionogel (S-ionogel) with a water content of around 34.55% by placing cellulose-CY colloid in an environment with a relative humidity of 55% to 60% for 36 h.

In addition, the Cel-gel was also prepared according to the above methods. Cellulose (1.579 g) and 30 g of [Bmim]Cl were mixed in a 3-neck flask and stirred at 80 °C for 4 h to form a homogeneous cellulose dissolving solution, noted as cellulose molecule/[Bmim]Cl. Then, the cellulose colloid was placed in an environment with a relative humidity of 55% to 60% for 36 h, obtaining the Cel-gel with a water content of around 30% to 40%.

### Determination of the SD

The SD value was calculated according to the following equation:

$$SD=\frac{\mathrm{Mm}\;*\;N\%}{100M_{N}\;-\;M_{\text{AN}}\;*\;N\%}$$ (1)

### Mechanical properties test

The Instron 5569 universal testing machine (Instron Corp., Canton, MA, USA) was used for tensile tests and cyclic mechanical testing. The impact resistance tests on S-ionogel, Cel-gel, and other samples were measured and calculated through the free-falling behavior of iron ball. The kinetic energy loss after the iron ball passed through the samples was used to determine the impact energy absorption of the samples. The energy absorption equation was calculated by the *m* (*V*_1_^2^ − *V*_2_^2^)/2*t*, where *m* is the mass of the iron ball (256 g); *t* is the thickness of the sample; *V*_1_ and *V*_2_ are the velocities of the iron ball before and after striking through the samples.

### Cytotoxicity test for human primary cardiomyocytes

The cytotoxicity test was conducted using the CCK8 method to detect the viability changes of HSF cells (primary cardiomyocytes, Primed-iCell-003, Shanghai Saibai Kang Biotechnology) and the samples (such as S-ionogel) co-cultured for 24 h. First, HSF cells were cultured in a special medium with 5% CO_2_ and 37 °C constant temperature incubator. The samples were then weighed, ultraviolet-sterilized, extracted at a concentration of 100 mg ml^−1^ for 24 h, and filtered through a 0.22-μm membrane to remove bacteria. The complete medium was diluted to working concentrations of 12.5, 25, 50, and 100 mg ml^−1^ for later use. The experiment was divided into 2 groups: Control and Sample 1 (S-ionogel). In the control group, 100 μl of complete medium was added per well; in the sample groups, 100 μl of sample working solution was added per well. Each treatment group had 3 replicate wells. Finally, the logarithmic growth phase HSF cells were counted, and the cell density was adjusted. Approximately 7×10^3^ cells were seeded in a 96-well plate. Following the grouping and treatment mentioned above, the cells were incubated in a 5% CO_2_, 37 °C constant temperature incubator for 24 h. After removing the culture medium, the wells were washed 3 times with PBS, and then 100 μl of medium containing 10% CCK8 was added per well. The cells were further incubated at 37 °C for 2 h in a 5% CO_2_, 37 °C constant temperature incubator. The absorbance value at 450 nm was measured using an enzyme immunoassay analyzer. The relative cell viability was calculated as follows: Relative viability% = (experimental group OD value − background OD value)/(mean OD value of control group − background OD value) × 100, where the background OD value was the absorbance of the medium with only CCK8 reagent and culture medium.

### Cytotoxicity test for human skin tissue cells

Human skin tissue cells (HaCat) were quickly removed from the liquid nitrogen tank and shaken in a 37 °C water bath until all melted. The cell suspension was quickly added to the prewarmed media and placed in a centrifuge at 1,000 rpm and centrifuged for 5 min. The centrifuge tube was removed, the supernatant was discarded, and fresh preheated medium was added to the tube. The cell suspension was added to a 100-mm culture dish and incubated at 37 °C with 5% CO_2_. When the cells are 80% to 90% full in the culture dish, digest the cells with 0.25% Trypsin EDTA, resuspend the cells with new medium, and passage at a ratio of 1:3 to 1:5 every 2 to 3 days. Take HaCat cells in logarithmic growth phase and in good growth condition, seed 4×10^3^ cells per well in a 96-well cell culture plate, and incubate overnight in a 37 °C, 5% CO_2_ incubator. Add 100 μl of sterile PBS to the wells around the cell wells.

The orifice plate was set up with the control group and experimental group: sample 1 (S-ionogel) at 200, 100, 50, 25, and 10 μg ml^−1^, with 3 replicate wells in each group. There were 2 control groups, 10 experimental groups, and 1 blank group in this experiment. The culture time was set at 24 h.

Absorbance values for each group were entered into Excel and the relative vitality was calculated (cell viability% = [experimental OD − background OD]/[control OD mean − background OD] × 100), where the background OD was the absorbance of the multiwell plate medium itself (wells with medium without cells).

### Thermal-electric response and signal test

#### Ionic conductivity measurement

First, place 2 nickel sheets with a width of 1 cm as current collectors on both sides of the samples. Then, connect the nickel sheets’ ends with platinum wires. Next, connect the setup to the CHI760e electrochemical workstation (Chenhua Instrument, Shanghai, China). Measure the AC impedance spectrum with an AC amplitude of 1 mV and a frequency range of 1 to 100 mHz. The sample’s ionic conductivity can be calculated using the formula: σ_i_ = *L*/(*R*_ion_ × *A*), where, *L* (cm) denotes the thickness of the sample, *A* (cm^2^) denotes effective cross-sectional area of the sample, and *R*_ion_ (Ω) denotes bulk resistance of the sample.

#### Thermoelectric voltage testing

Prepare an S-ionogel sample (3 cm × 3 cm ×1 cm) and place 2 platinum sheets (3 cm × 3 cm) as current collectors on both sides of the sample. Then, connect the sample setup to the CHI760e electrochemical workstation (Chenhua Instrument, Shanghai, China). Constructing a thermal gradient for the testing process: we place the complete sample assembly on an electric heating plate or human wrist and chest, where the contacting surface of S-ionogel as the hot end and the other end exposed to air as the cold end. Using the 2 thermocouples, measure the temperature (*T*_1_, *T*_2_) at both ends of S-ionogel. The corresponding open circuit voltage of *V*_1_ and *V*_2_ was measured using the electrochemical workstation. The sample’s ionic Seebeck coefficient can be calculated using the formula: *S*_i_ = (*V*_2_ − *V*_1_)/(*T*_2_ − *T*_1_).

#### Sensing signals monitoring test

The self-powered S-ionogel was connected to a CHI760e electrochemical workstation (Chenhua Instruments, Shanghai, China). The current waveforms of e-skin sensing external stimuli of heart vibrations, touch, and temperature changes were assessed by measuring the amperometric *I–t* curve parameters with an initial *E* of 0 V at room temperature.

## Data Availability

All data supporting the findings of this study are available within the paper and its Supplementary Materials. In addition, the datasets generated or analyzed during this study are available from the corresponding authors on reasonable request.

## Supplementary Materials

Supplementary 1 Notes S1 and S2 Figs. S1 to S19 Movies S1 to S3
