# Supplementary material for: A Cellulose Ionogel with Rubber-Like Stretchability for Low-Grade Heat Harvesting
Source: Research (Wash D C). 2024 Nov 18;7:0533. doi: 10.34133/research.0533 (PMC11570788; doi:10.34133/research.0533)
Supplement: Supplementary 1 — Notes S1 and S2 Figs. S1 to S19 Movies S1 to S3 [file research.0533.f1.zip › Revised Supporting Information (Clean Version).docx]

**Supplementary Materials**

**A cellulose ionogel with rubber-like stretchability** **for low-grade heat harvesting**

Qian Long^1,#^, Geyuan Jiang^1,#^, Jianfei Zhou^3,*^, Dawei Zhao^1,2,*^, Haipeng Yu^2,*^

1. Key Laboratory on Resources Chemicals and Materials of Ministry of Education, Shenyang University of Chemical Technology, Shenyang, China
2. Key Laboratory of Bio-based Material Science and Technology of Ministry of Education, Northeast Forestry University, Harbin, China
3. College of Biomass Science and Engineering, Sichuan University, Chengdu, China

^#^ Q.L. and G.J. contributed equally to this work.

* Corresponding to:

[syzhaodawei2014@nefu.edu.cn](mailto:syzhaodawei2014@nefu.edu.cn);

[yuhaipeng20000@nefu.edu.cn](mailto:yuhaipeng20000@nefu.edu.cn);

[zhoujf2014@scu.edu.cn](mailto:zhoujf2014@scu.edu.cn);

**Note S1. Characterization methods of S-ionogel**

The chemical structures of cellulose and cellulose-CY were subjected to solid-state ^1^H NMR analysis at 25 ℃ using a Bruker 600M (Bruker, Germany). Analysis of CHNSO elements was carried out using the Vario EL Cube instrument (Elementar, Germany). The Nicolet IS50 spectrometer (Thermo Scientific Nicolet IS50 USA) was employed to measure the FTIR spectra of the samples. Spectra were collected across the range of 400−4000 cm^-1^. Microscopic SEM images of the S-ionogel, Cel-gel and others were investigated by a JSM-IT800 scanning electron microscope (Hitachi, Tokyo, Japan) at an operating voltage of 20 kV. Light transmittance (T) and haze values of the samples were tested by a Jindou (JDY200). The X-ray diffraction of the samples was tested by a Rigaku D/Max-Ultima^+^, Japan, with a test angle of 5-40 °. The SAXS of samples was measured using an SAXSess mc2 instrument with Cu-Kα X-ray radiation and a wavelength of 0.154 nm (Anton Paar, Graz, Austria). The sample-to-detector distance was 2658.5 mm and exposure time 5 min.

**Note S2. The molecular dynamics (MD) simulations of cellulose and cellulose-CY**

According to the experimental conditions, two systems were constructed (System 1 is cellulose; system 2 is cellulose-CY). In the [Bmim]Cl and H_2_O solution system, both cellulose and cellulose-CY consisted of 10 chains, with each chain consisting of 10 monomers. The initial configurations of the systems were constructed using the Packmol software. The simulations were performed using GROMACS package (version 2019.3) with the all-atom OPLS (optimized performance for liquid systems) force field. For each system, the steep descent method was used to minimize the energy of the system. Then, molecular dynamics simulations under NPT ensemble were performed for 10 ns for each system. LINCS algorithm was applied to constrain the bond lengths of other components. Periodic boundary conditions were applied in all three directions. The temperature was maintained using the V-rescale thermostat algorithm. The pressure control was changed to the Parrinello-Rahman method in the production run. The cut-off distance for the Lennard-Jones and electrostatic interactions was 1.2 nm. Particle mesh Ewald method was used to calculate the long-range electrostatic interactions. Configurations were visualized using Visual Molecular Dynamics software.


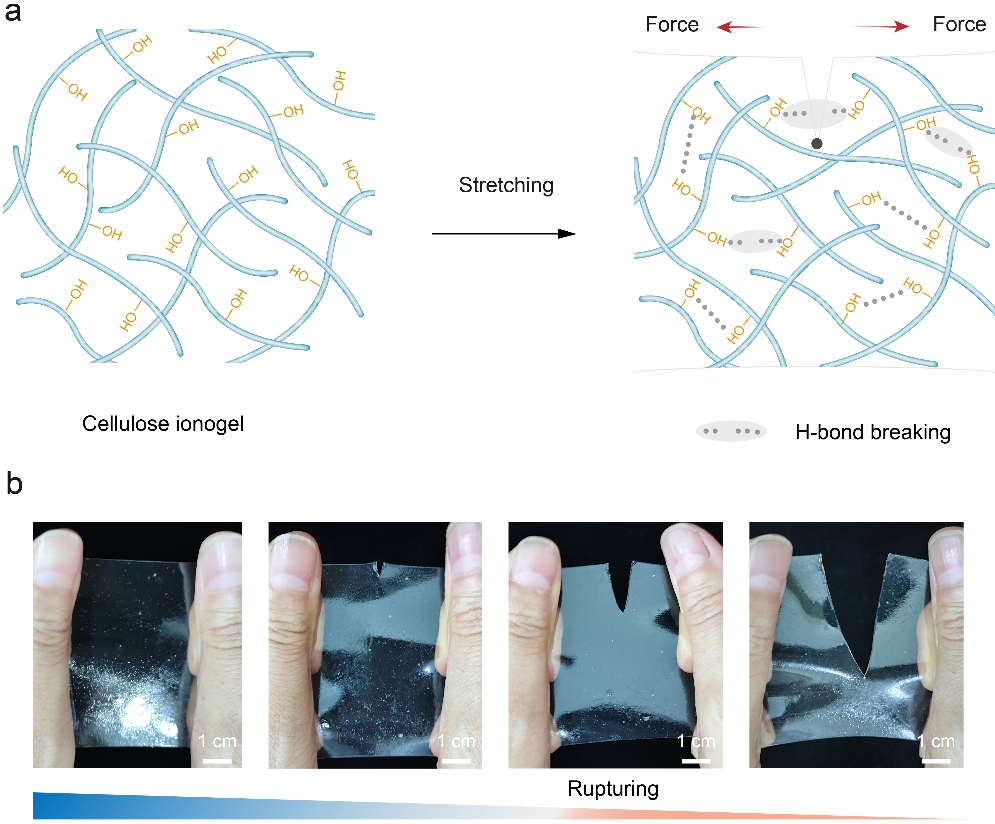


**Figure S1.** Molecular network and performance of Cel-gel. **a**, The changes of molecular network of Cel-gel during stretching process. **b**, Optical images of Cel-gel showing the rupturing structure when treated by external forces.


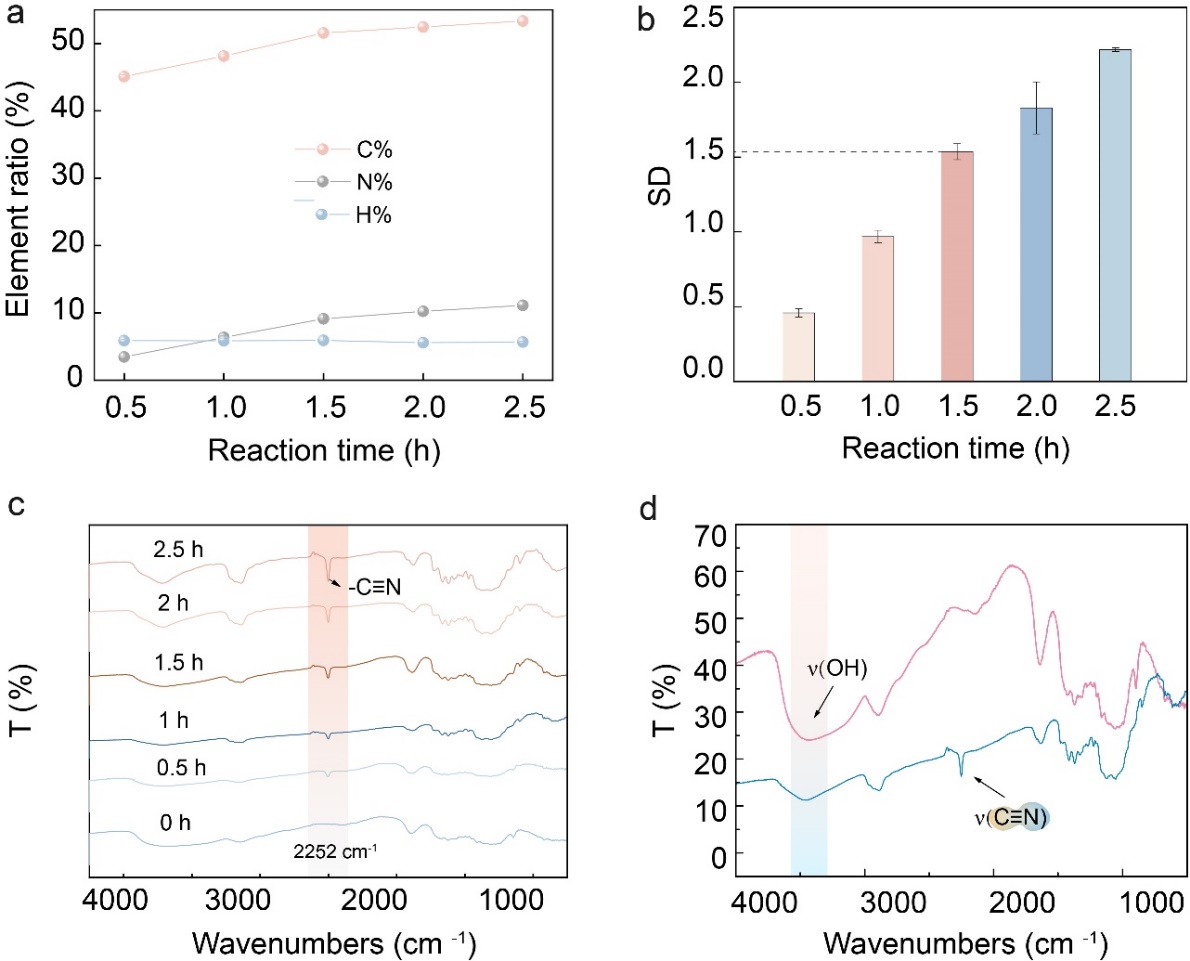


**Figure S2.** Quantitative analysis and spectrum analysis of cellulose and cellulose-CY. **a**, Investigating the element ratio of cellulose-CY during the different cyanation treatment time. **b**, Calculated the SD of cellulose-CY molecule during the different cyanation treatment time. **c**, FTIR spectra of cellulose-CY during the different cyanation treatment time. **d**, Compared the FTIR spectra of S-ionogel and Cel-gel.


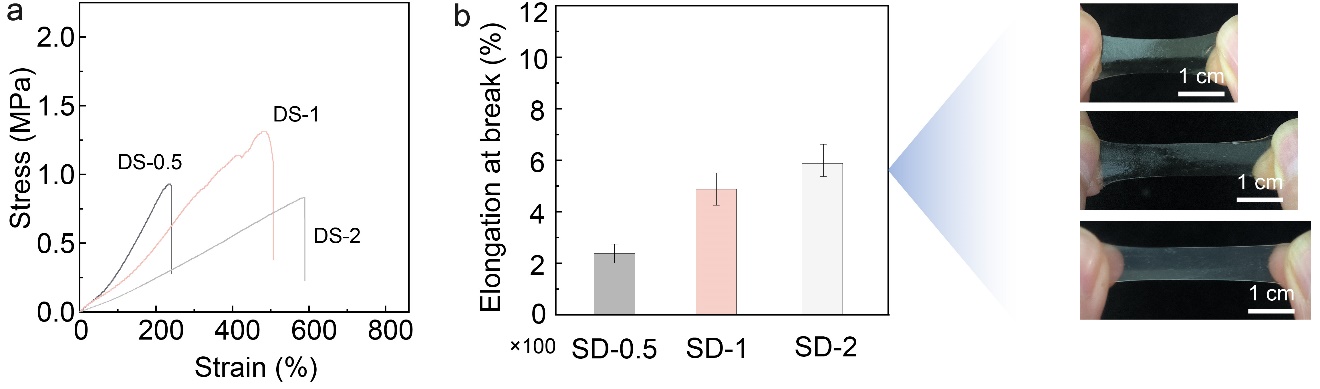


**Figure S3.** Investigating the mechanical properties of S-ionogel. **a**, Tensile stress–strain curves of the S-ionogel with SD of 0.5, 1 and 2. **b**, Comparison of the elongation at break between S-ionogel with SD of 0.5, 1, and 2. Optical images show the stretchability of S-ionogel.


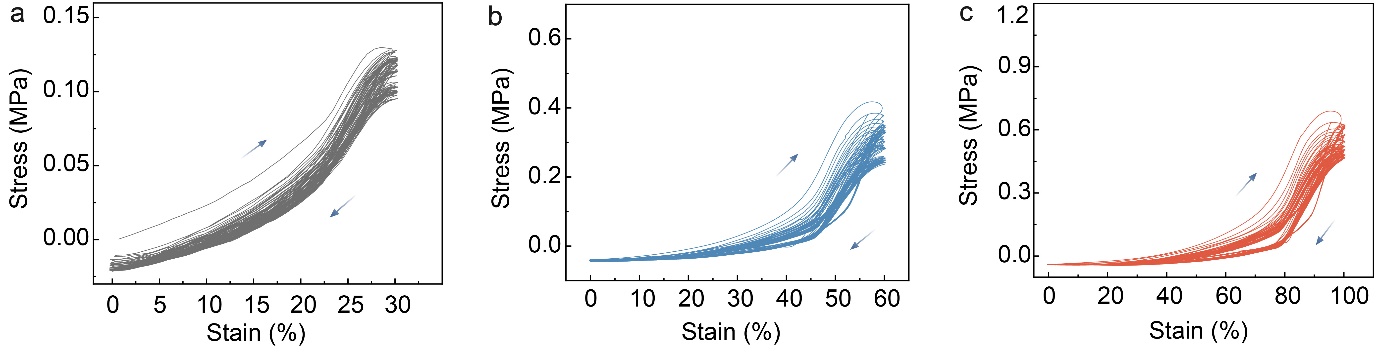


**Figure S4.** Repeated tensile stress-strain curves for the S-ionogel. **a**, The 30 cycles of tensile curves of S-ionogel at 20% strain. **b**, The 50 cycles of tensile curves of S-ionogel at 60% strain. **c**, The 50 cycles of tensile curves of S-ionogel at 100% strain.


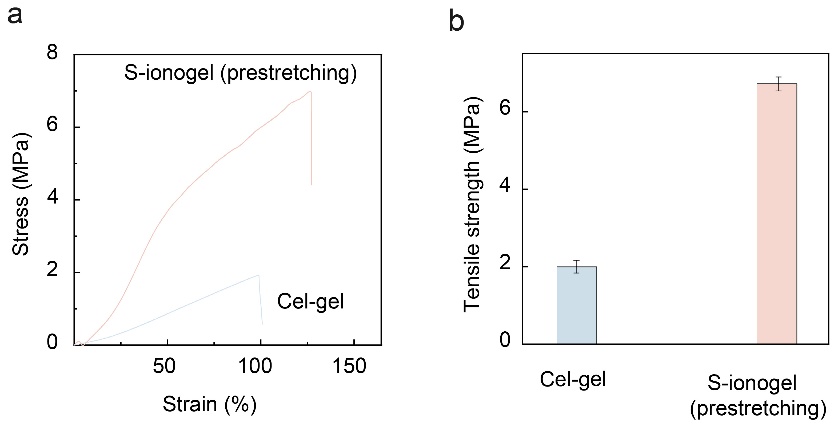


**Figure S5.** Investigating the mechanical properties of S-ionogel. **a**, Tensile stress–strain curves of S-ionogel (pre-stretching for 80% strain) and Cel-gel. **b**, compared the tensile strength of Cel-gel and S-ionogel (pre-stretching).


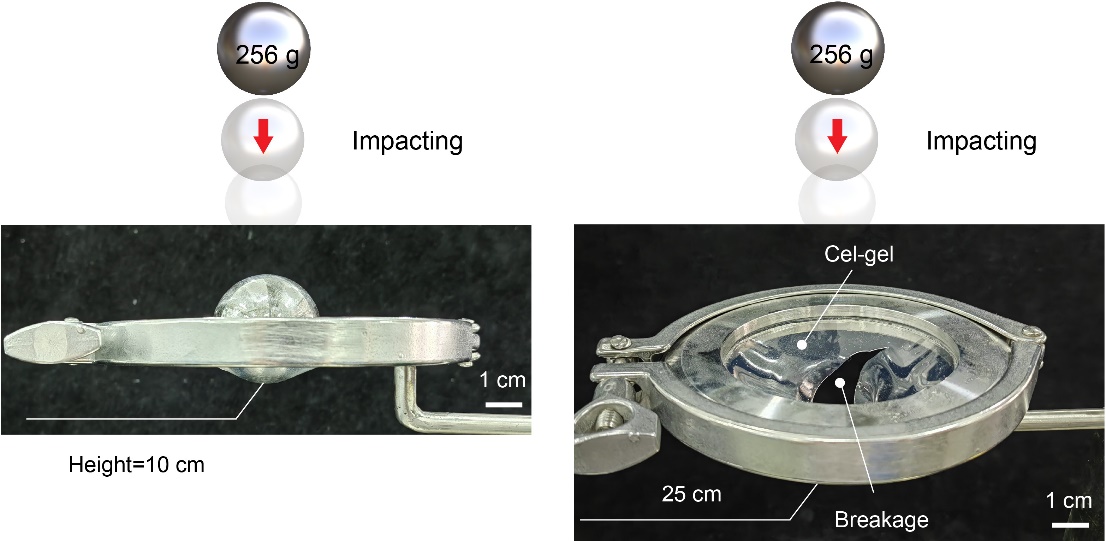


**Figure S6.** Free-fall impact resistance of the Cel-gel. The weight of the iron ball was 256 g. The optical images show that the Cel-gel is badly breakage.


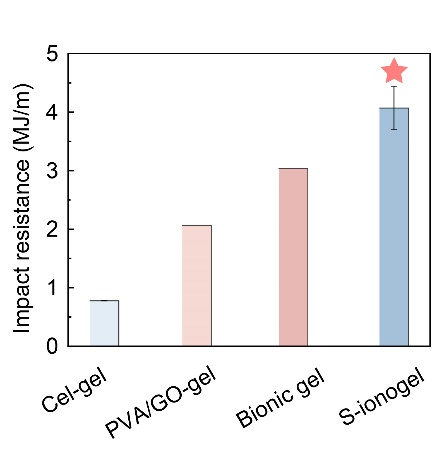


**Figure S7.** Compared the impact resistance of Cel-gel, PVA/GO-gel, bionic gel, and our S-ionogel.


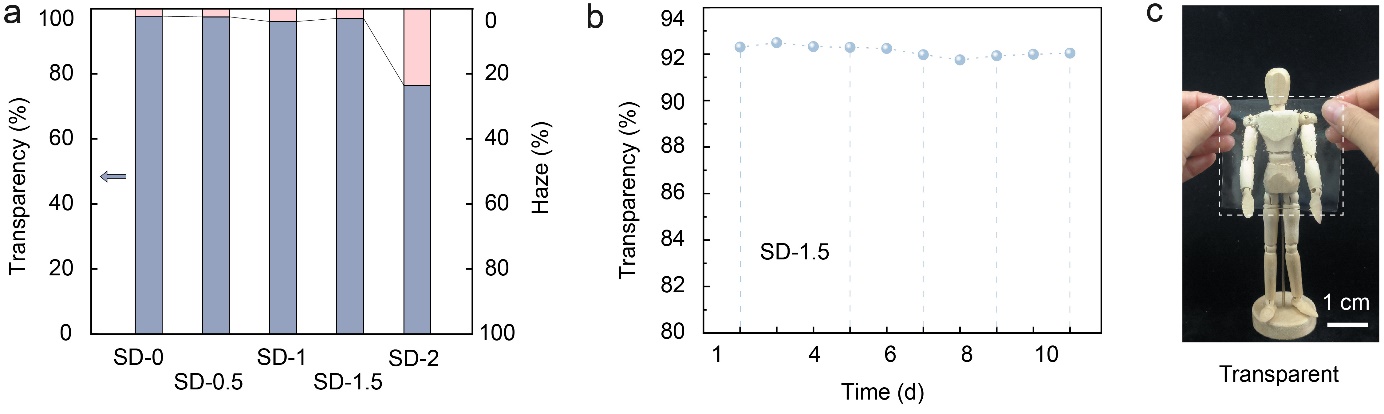


**Figure S8.** Investigating the light properties of S-ionogel during the different cyanation treatment time. **a**, Studying the transparency and haze of S-ionogel with different SD. **b**, Investigating the optical stability and high transparency of S-ionogel. **c**, Optical image of S-ionogel, showing good transparency.


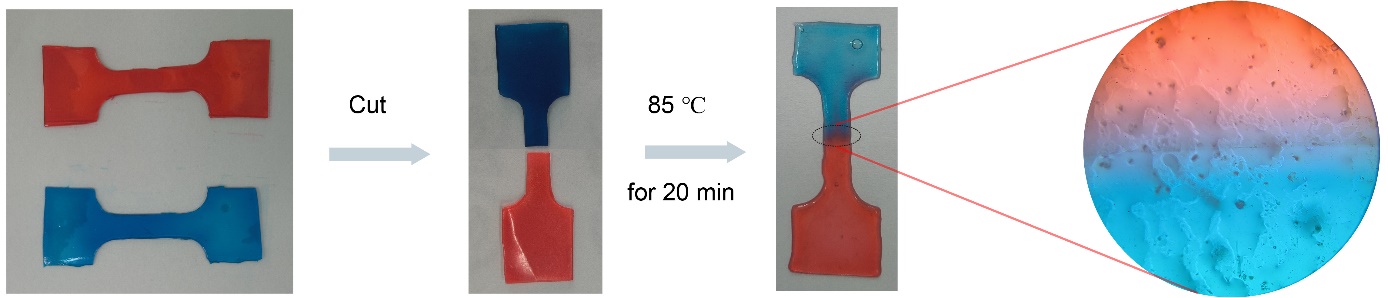


**Figure S9.** Investigating the self-healing behavior of S-ionogel.


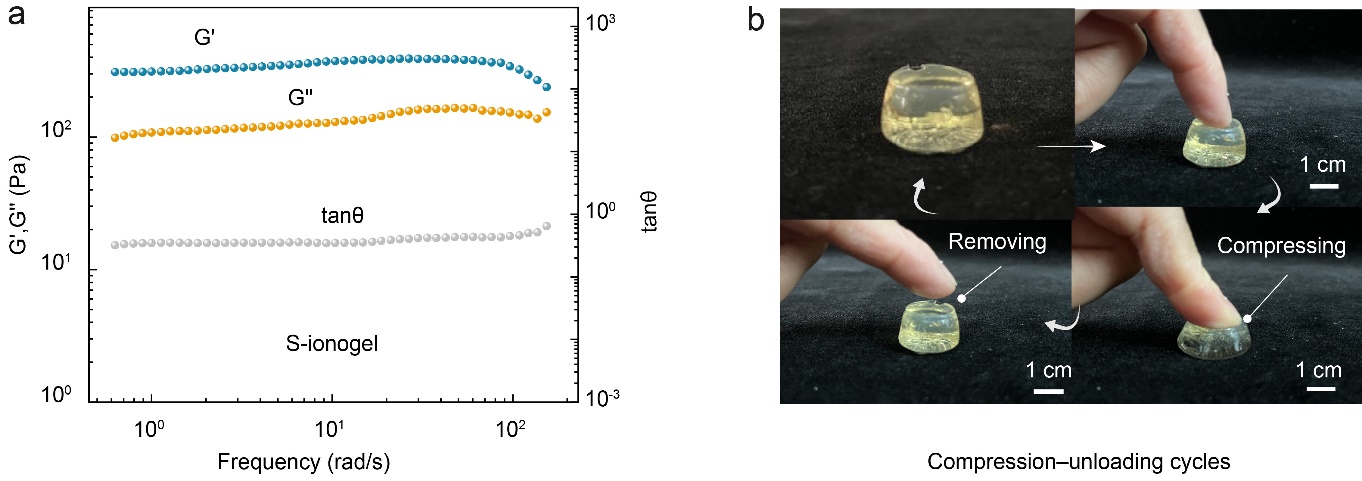


**Figure S10.** Investigating the viscoelastic behavior of the S-ionogel. **a**, Viscoelastic behavior of S-ionogel. G' is also known as the elastic modulus, that reflects the elastic properties of material. G'' also called as the viscous modulus reflects the viscous properties of material. tanθ is called damping factor. **b**, Optical images of S-ionogel showing good resilient behavior during compressing and removing process.


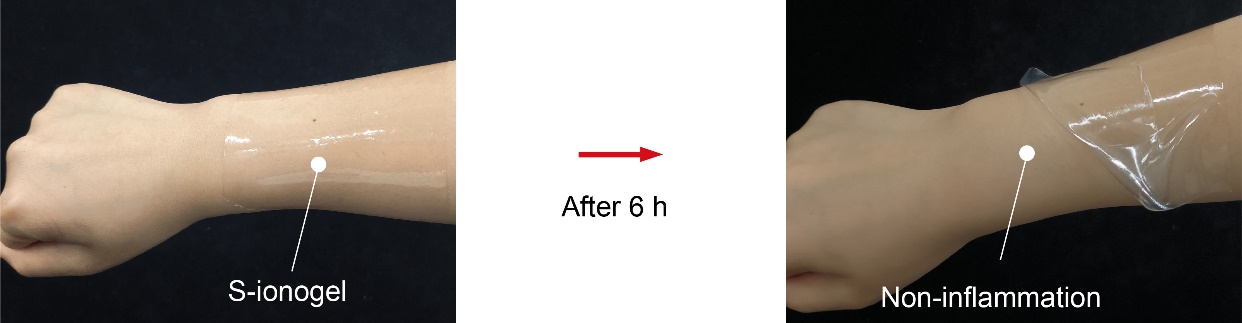


**Figure S11.** Optical images of S-ionogel placing in human wrist for 6 h, showing good biocompatibility.


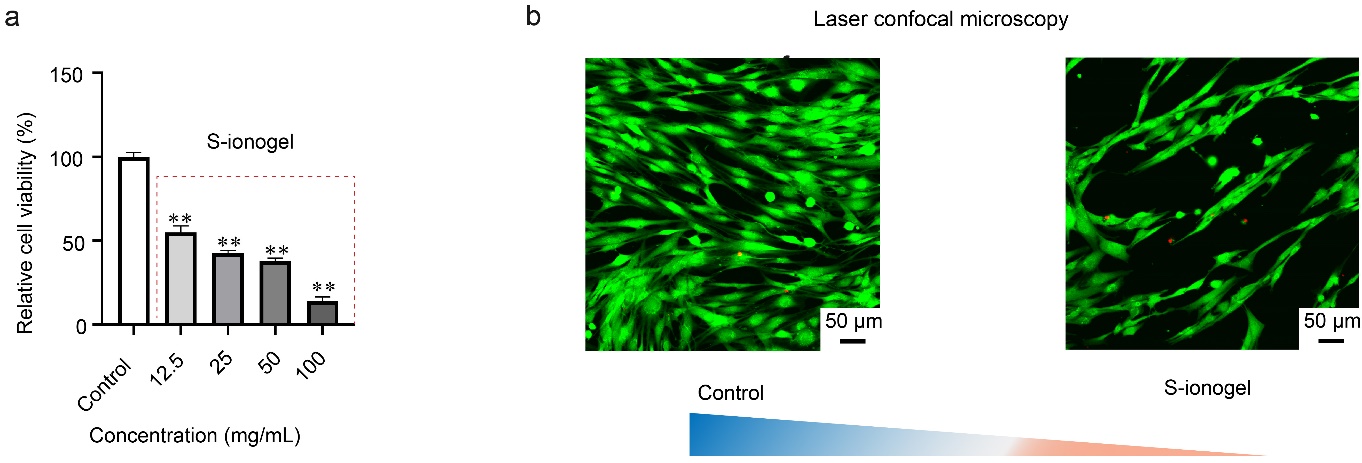


**Figure S12.** Investigating the cytotoxicity of S-ionogel. **a**, Cytotoxicity test of the S-ionogel. **b**, Live/dead staining confocal images of S-ionogel and HSF cells co-cultured for 24 h (scale bar is 50 μm).


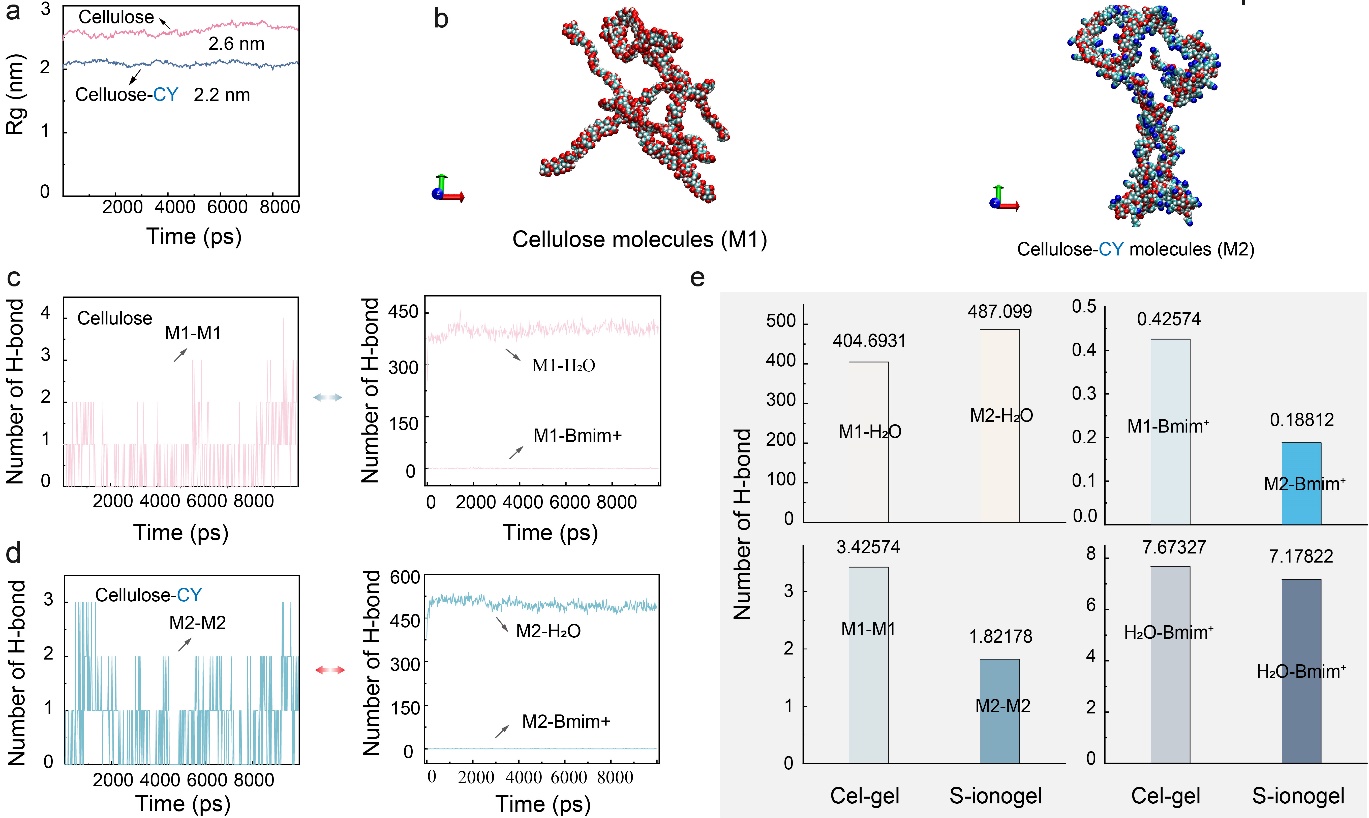


**Figure S13.** Molecular dynamics (MD) simulation of cellulose and cellulose-CY. **a**, The radius of gyration (R_g_) of cellulose and cellulose-CY. **b**, MD simulation of cellulose and cellulose-CY molecular chain. **c**, Investigating the number of H-bond between cellulose molecule with other components. **d**, Investigating the number of H-bond between cellulose-CY molecule with other components. **e**, Comparation the number of H-bond between Cel-gel and S-ionogel.


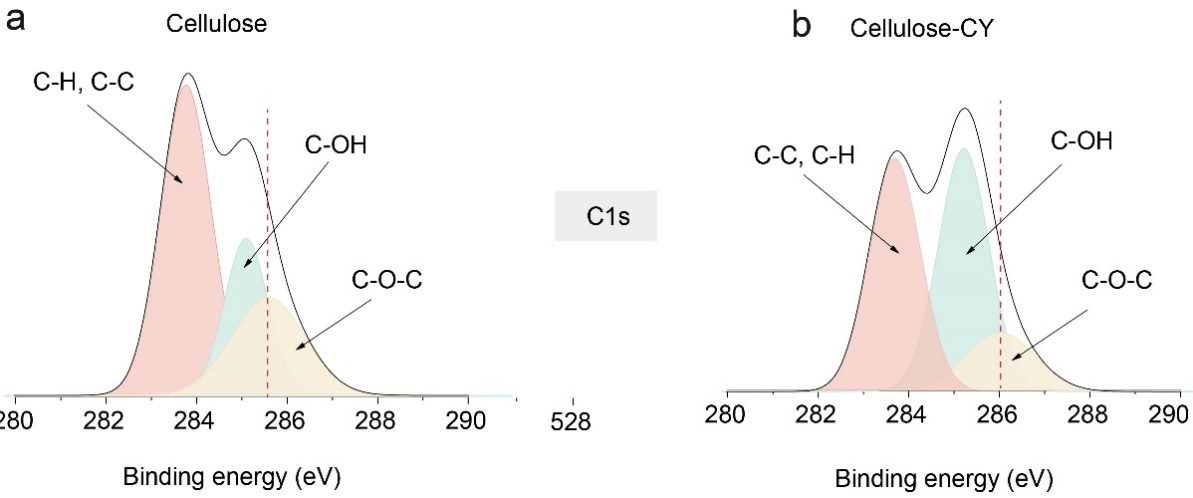


**Figure S14.** XPS spectra of cellulose **a** and cellulose-CY **b**.


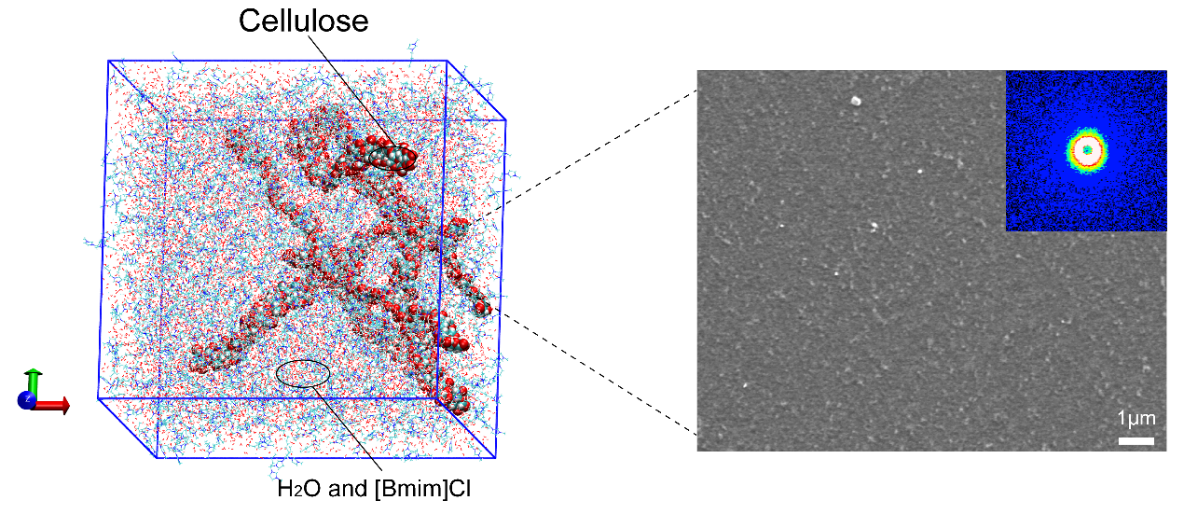


**Figure S15.** Snapshots of cellulose molecules with [Bmim]Cl and H_2_O. The colored spherical chains represent cellulose molecule and the rest of chains represent the [Bmim]Cl and H_2_O. SEM images and SAXS 2D patterns of Cel-gel.


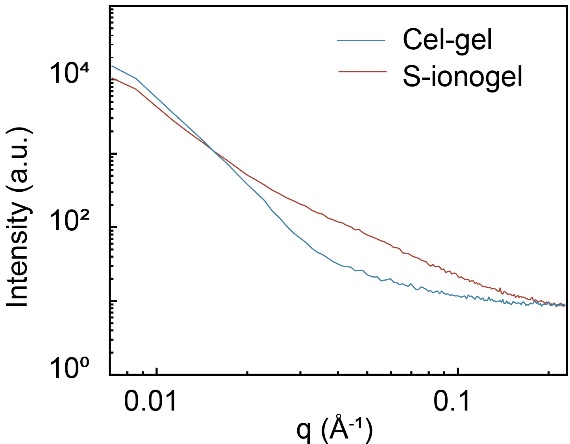


**Figure S16.** Investigating the SAXS curves of Cel-gel and S-ionogel.


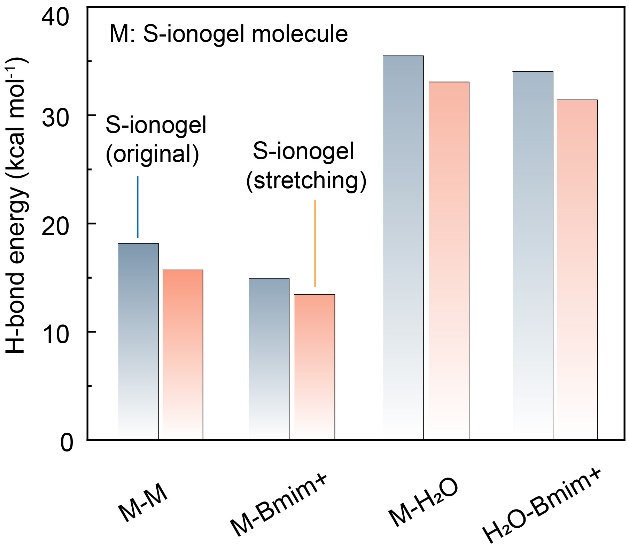


**Figure S17.** Investigating the H-bond energy from MD simulation of S-ionogel during different state.


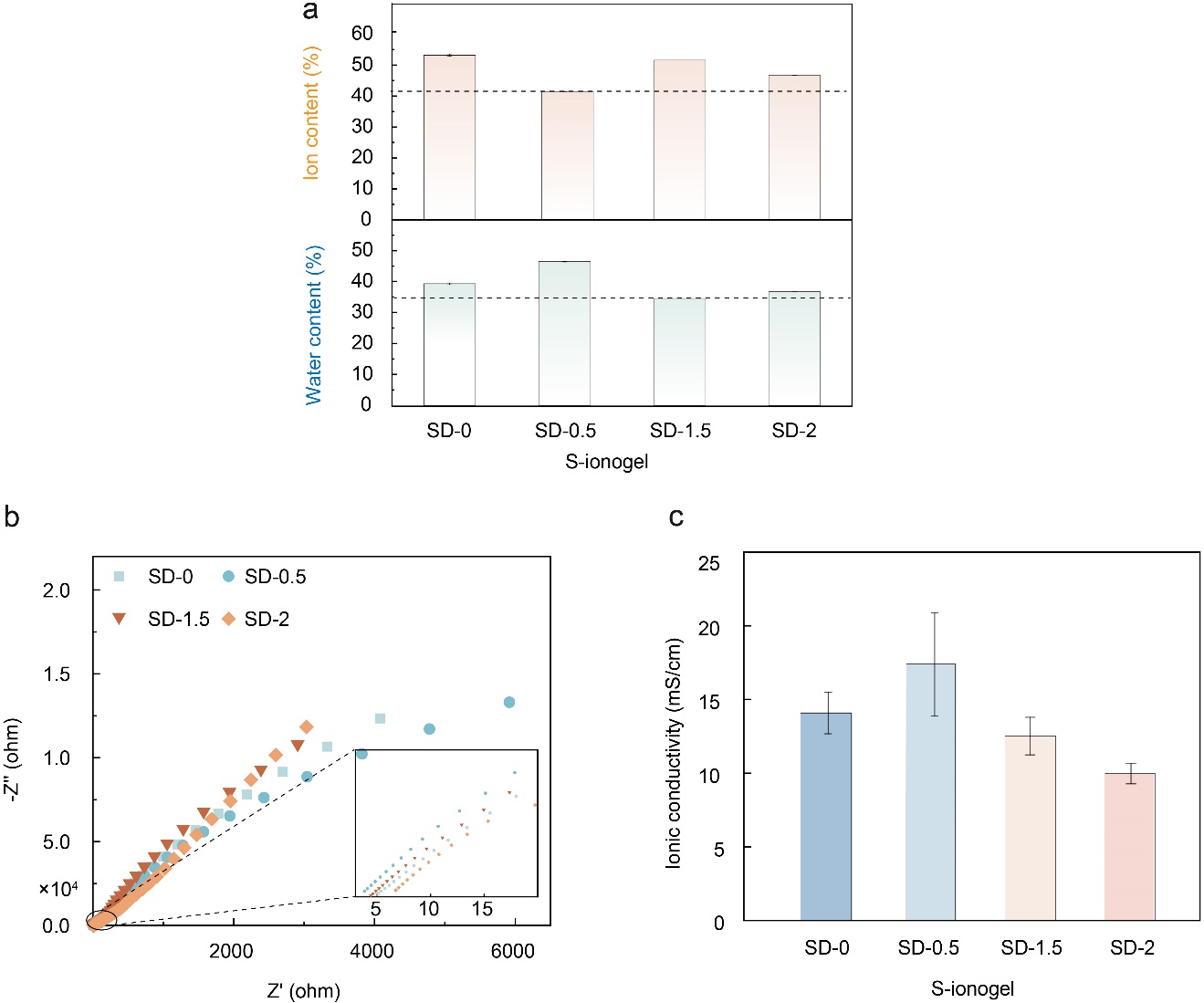


**Figure S18.** Investigating the ionic conductivity of S-ionogel. **a**, The H_2_O and ion contents of S-ionogel during different SD. **b**, Electrochemical impedance spectroscopy curves of S-ionogel with different SD. **c**, Comparison of the ionic conductivity of S-ionogel with different SD.


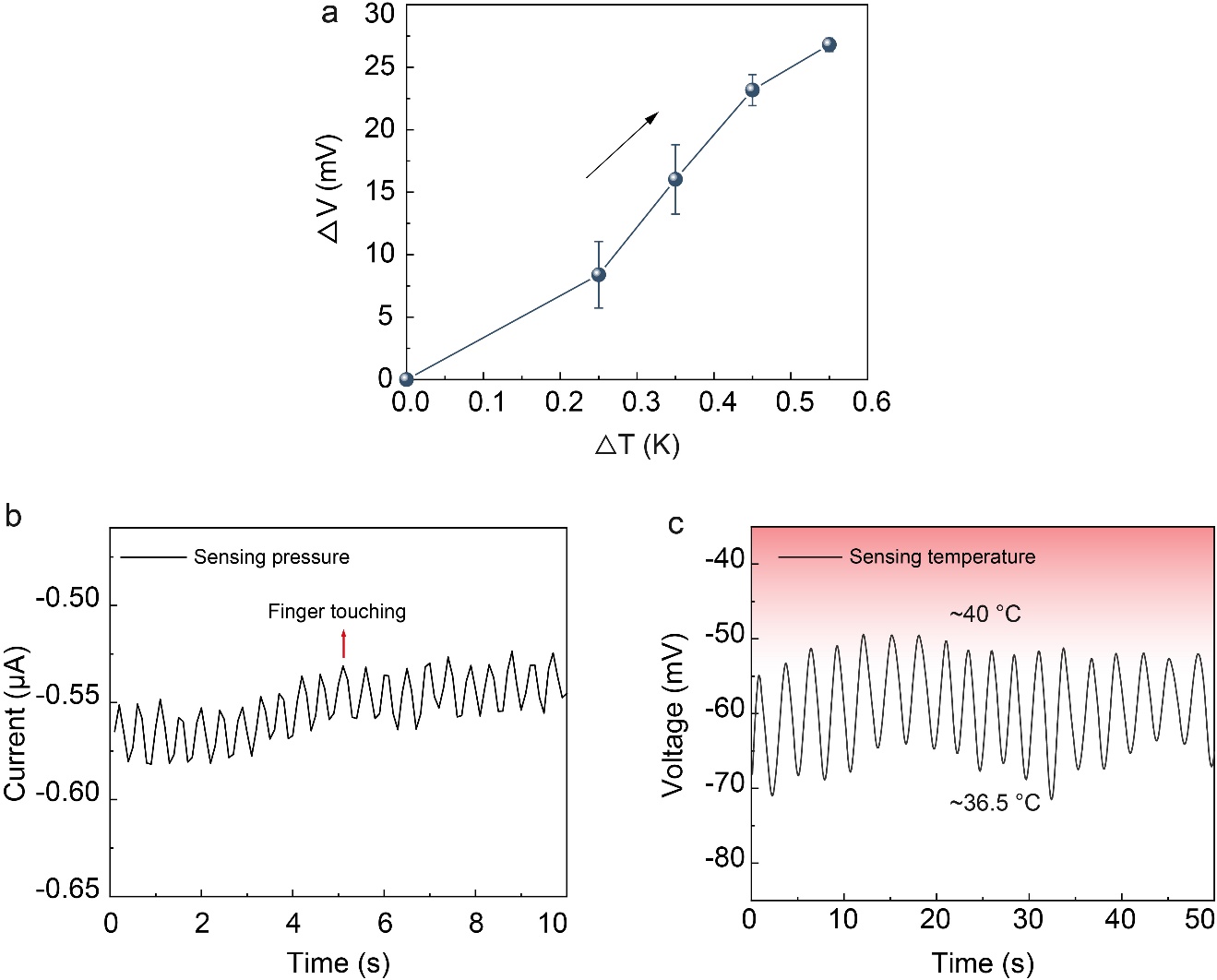


**Figure S19.** Self-powered S-ionogel-based e-skin. **a**, Function diagram of ∆V and ∆T, showing the good thermal-electric response. **b**, Self-powered e-skin detecting the current signals caused by finger touching. **c**, Self-powered e-skin responds to the changes in temperature.
